# Multiscale modeling in the framework of biological systems and its potential for spaceflight biology studies

**DOI:** 10.1016/j.isci.2022.105421

**Published:** 2022-10-26

**Authors:** Andrew Millar-Wilson, Órla Ward, Eolann Duffy, Gary Hardiman

**Affiliations:** 1 MSc Bioinformatics and Computational Genomics, Queen’s University Belfast (QUB), Belfast, NI, UK; 2 Faculty of Medicine, Health and Life Sciences, School of Biological Sciences and Institute for Global Food Security (IGFS) Queen’s University Belfast (QUB), Belfast, NI, UK

**Keywords:** systems biology, in silico biology, space sciences

## Abstract

A central tenet of systems biology is that biological systems are greater than the sum of their component parts. Spaceflight is associated with hazards including radiation exposure and microgravity which impact different echelons of biological organizations spanning molecular, cellular, organ, and organismal levels. These insults lead to physical damage, including muscle and bone loss, neurological damage, and impaired immunity. Mitochondrial dysfunction and biological alterations occurring during spaceflight have been reported. The health challenges presented by long-term space travel must be addressed and appropriate countermeasures developed to protect astronauts. Increasing quantity of multiomics data are being generated from cells and model organisms flown in space, with physiological data from astronauts. Systems biology approaches leveraging mathematical reasoning and computational modeling are required to characterize these components in a holistic fashion. In this review, we provide an historic perspective on multiscale biological systems modeling, followed by a discussion on its utility for spaceflight biology research.

## Graphical abstract

**Figure:**
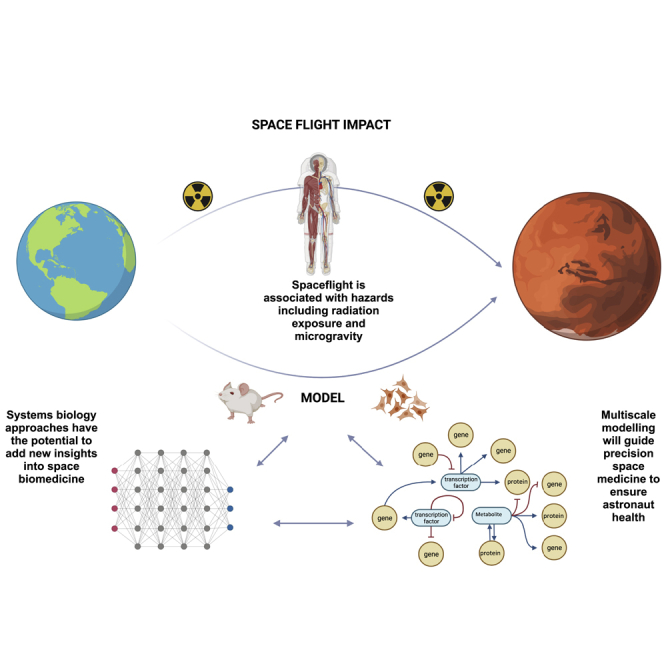

## Introduction

**Figure 1:**
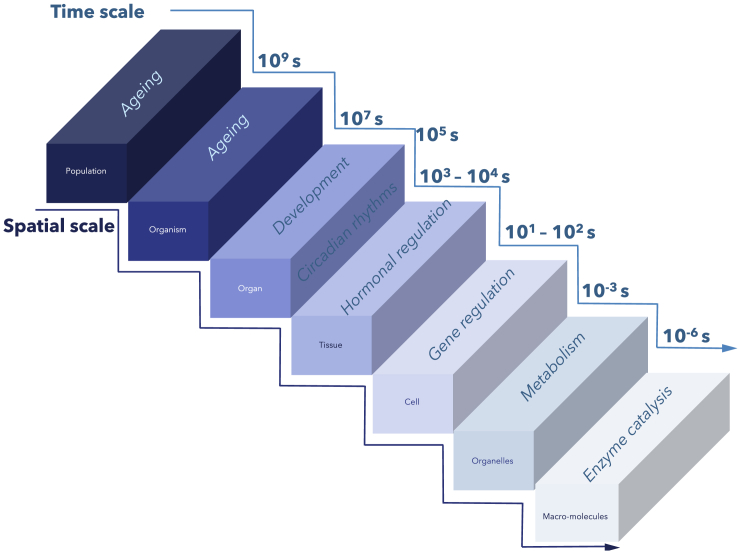
Spatial and temporal scales in biology Temporal scales range from short such as enzyme catalysis (10^-^^6^) to long such as development or aging (10^9^ s). Spatial scale ranges from macromolecules, organelles, and cells to organisms and populations.

Systems biology uses mathematical reasoning, high-throughput techniques, and computational modeling^1^ to characterize biological entities or systems in a holistic fashion by studying their constituent processes at various spatiotemporal scales.^2^^,^^3^^,^^4^ A central tenet of systems biology is that biological systems are more than the sum of their parts,^5^ thus the holistic behavior cannot be understood by studying 1 element of these interconnected systems in isolation. The scales at which these elements exist are diverse. Spatially, they range from 10^−10^ m at the molecular scale to meters at the organismal scale and beyond for ecological interactions. Temporally, they range from nanoseconds to years,^2^ with a positive correlation between an increased spatial level and an increased temporal level, i.e., lower-spatial-scale processes occur at a faster speed than those at a higher scale.^6^ To analyze such disparate scales, systems biology investigates the flow of information from subcellular components and their encoding genes and proteins and their related biological pathways that regulate organism-level functions within organs, tissues, and cells (Figure 1).^7^

Experimental-data-based computational and mathematical models have been utilized to understand and predict the behavior of these systems,^5^ making systems biology an inherently interdisciplinary field. Although biological modeling is not a novel idea, simple modeling techniques like differential equations or Boolean networks are unable to model these complex systems, and multiscale modeling is required.^4^ As such, the popularity of systems biology is a postgenomic phenomenon with genomic era advancements facilitating systems biology modeling, for example, (1) development and availability of high-throughput omics techniques^8^; (2) widespread omics data availability through online repositories; (3) increasing popularity of interdisciplinary science leading to new approaches in computational biology, network analysis, programming, and ontologies^9^^,^^10^; and (4) advances in internet and computational infrastructure allowing easier data dissemination.^3^

Spaceflight is associated with hazards such as radiation, microgravity, and isolation which impact astronauts at multiple levels of biological organization from genome to proteome.^11^^,^^12^ These impacts precipitate numerous ill-health outcomes including physical damage, such as muscle and bone loss, neurological damage, and impaired immune function,^11^^,^^13^ and psychological stress leading to depression, feelings of loneliness, and psychosomatic disorders.^14^ Research to ameliorate these impacts has already found use on Earth, with USSR cosmonaut training vibration platforms being used to treat old-age muscular atrophy. However, given the rise in private spaceflight and the involvement of the private sector in government spaceflight, it is imperative that this research remains as open as possible and that the multiomics data generated on these missions be freely available for the advancement of both terrestrial and spaceflight research,^15^^,^^16^ including the development of multiscale biological models based on these data. In this review, we provide an historic perspective on multiscale biological systems modeling, followed by a discussion on its utility in spaceflight biology research.

### Multiomics integration is essential to systems-level analysis

Biological systems operate at multiple spatiotemporal scales.^2^ Models incorporating multiple scales are significantly more complex than single-scale models because links between the different scales must be defined and quantified. Furthermore, as more components, parameters, and relationships are incorporated into a model, it becomes more difficult to balance accuracy and computational feasibility. Since behavior at the smaller scales of a system can influence the behavior at larger scales, it is necessary for a link between these scale levels to be incorporated into the multiscale model. Simultaneously, the scales that define the modeled levels are dependent on the process under investigation. It can sometimes be appropriate to combine 2 levels/scales (for example, cellular and multicellular) or to split a level into distinct sublevels to create a more comprehensive or accurate model.^6^ In other words, the creation of the model should be tailored to the process in question to optimize simulation and predictive power.

Different biological scales can be represented by different omics outputs, for example, genetic scale interactions using genomics and epigenomics, expression-level interactions by transcriptomics, followed by proteomics and metabolomics for protein scale interactions and their products.^17^^,^^18^^,^^19^^,^^20^^,^^21^ This highlights that to represent biological entities in a holistic fashion in keeping with systems biology, multiomics techniques must be utilized.^2^^,^^10^^,^^22^ Utilization of multiomics data bolsters single-omics methods, helping to put results into context. For example, the network-assisted genomic analysis (NAGA) approach builds on the availability of genome-wide association studies (GWAS), which associate single-nucleotide polymorphisms (SNPs) with the disease using appropriate measures of statistical significance. NAGA places these results in the context of protein interaction networks, giving greater weight or importance to SNPs appearing on the same network. This technique allows disease-associated genes that would not otherwise meet the prohibitively stringent p value cutoff required by GWAS to be considered.^23^

Multiomics datasets can be integrated in various ways, and some of them are discussed in the following list:

1. Postanalysis integration
  Omics datasets are analyzed separately; features of interest are isolated and subsequently used to form nodes connecting datasets in an overall network. This approach has been exploited in a diverse set of studies, including for the assessment of biological wastewater treatment systems,^24^ the exploration of microbial resistance of marine sediments following an oil spill,^25^ and studying the permafrost microbial ecosystem.^26^
2. Integrated data analysis
  Different omics datasets are integrated using tools, such as multiple-block orthogonal projections to latent structures prior to the analysis. The advantage of this approach is that key similarities between omics datasets are uncovered using mathematical rather than interpretational approaches, thereby reducing operator bias. This approach was successfully exploited in a study of 22 asthmatics and healthy controls. A joint data structure in 6 data blocks was interrogated:transcriptomics; metabolomics; targeted sphingolipids, oxylipins, and fatty acids assays; and a clinical block encompassing lung function, immune cell differentials, and cytokines. The model uncovered 7 components of which components 1 and 2 were the most useful, identifying differences between healthy controls and asthmatics and a disease-sex interaction, respectively.^27^
3. Systems modeling techniques
  These facilitate data analysis and prediction of system behavior. Extensions from single-cell to multicellular systems and multiorgan systems commenced in 2013 with the development of Recon 2, community-based global reconstruction of human metabolism.^28^ This model incorporates cell- and tissue-specific metabolomics data, proteomic data, and gene expression data for the human body and its many cell types. The main limitation is that the modeling often requires a deep understanding of the modeled system, so new experimental findings can be properly compared to model predictions.^29^

Due to the predictive abilities of systems modeling approaches,^5^ from this point, we will focus on systems modeling approaches.

### Systems modeling basics

Biological systems exist on multiple levels of a mutually interacting organization; therefore, to accurately represent these systems, models must be multilevel with both upward (i.e., how the components affect the overall system) and downward (i.e., how the overall system behavior affects each component) relationships. Such models will likely be hybrid and integrate multiple models.^5^

On a gross scale, 3 methods can be exploited to model a biological system:

1) Top-down
  This method helps to analyze the overall system behavior to generate hypotheses about and explore system components. Although this approach is easier than others, its variables can become detached from biological reality, and it lacks the flexible and robust nature of bottom-up approaches.^2^
2) Bottom-up
  This method helps to study individual system components and their interactions, explaining system behavior as an emergent property. This allows new information to be added easily, making bottom-up models very robust; however, they often have prohibitively high computing power requirements.^2^
3) Middle-out
  Finally, middle-out approaches first model an intermediate scale such as individual cells. They are then expanded, analyzing smaller and larger scales.^2^^,^^4^

The development of a complex model inevitably involves making assumptions, which are necessary to simplify the model. In doing this, it is important to keep in mind that the solutions produced are approximations and that more valid assumptions produce more accurate results. The overall goal of multiscale modeling is to obtain the highest possible level of accuracy and predictive power despite introducing constraints required to facilitate working with the data and minimize the time required to produce results. Given the complexity and magnitude of multiscale modeling, it is often dependent on computational methods to obtain solutions.^30^

### Historical approaches to multiscale modeling

Several multiscale modeling approaches have been developed to date to model biological systems. The choice of method depends upon the range of spatial and temporal scales being investigated. When creating a model for multiple scales, the individual scale models could be combined explicitly, or alternatively, the output from one model could be used as an input for another. In the latter case, chemicals in a reaction could be assumed to be in equilibrium if the timescale is very fast, or boundary/initial conditions in terms of a smaller scale model could be used in a model of a larger scale.^30^ The quasicontinuum method is used at the atomic spatial scale and involves coarse graining, a method which greatly reduces computational cost. Coarse graining involves selecting a subset of the total number of atoms (known as the "representative atoms") to model as individual atoms, while the assumption of continuum mechanics is used to model the rest of the atoms. In doing this, the degrees of freedom and the computational demand are lowered.^4^

A molecular-level-modeling method is a hybrid of quantum mechanics-molecular mechanics (QM/MM), which uses quantum mechanical calculations for areas involved in chemical reactions and molecular mechanics for all other areas. An example of the use of this model would be modeling an enzyme, where a quantum mechanical model would be used for the catalytic site on the enzyme while a molecular mechanics model would be used to describe the interaction of the enzyme with its surrounding environment.^4^

The equation-free multiscale method allows a model at the microscopic level to influence modeling at the macroscopic level. It does not require the derivation of macroscopic evolution equations, as the name suggests, and there are several techniques involved: coarse projective integration, gap-tooth scheme, and patch dynamics.^4^^,^^31^ Coarse projective integration involves the calculation of time derivatives for coarse variables of microscopic solutions, which are then used to estimate the coarse variable over a bigger time step.^4^ The gap-tooth scheme approximates a time derivative of a macroscopic equation that is unavailable in a macroscopic domain. It does this by using simulations of the microscopic model that are available, in several small boxes that only span part of the domain.^31^ Meanwhile, patch dynamics is a combination of coarse projective integration and the gap-tooth scheme. It approximates the behaviors at the macroscale by computing the microscale dynamics and confining them to a subset of patches. Here, a patch is defined as a small region over a short time period.^4^^,^^32^ The heterogeneous multiscale model (HMM) starts off with a macroscale solver and, taking the provided information about the macroscale process into account, uses a microsolver to fill in the gaps in the macroscale data. HMM is dependent on a coherent link between the macroscopic and microscopic models.^4^

The multigrid method is a way to solve a variety of integral and partial differential equations (PDEs) rapidly and efficiently. It is also capable of considering separate natures of models at various scales.^4^ The multiscale agent-based-modeling (MS-ABM) method is the only method described here that uses a middle-out approach, and it can be used at any intermediary level between molecular and population levels. It is particularly good at simulating the behavior of a system using rules and interactions within the individual components. However, this method does require a lot of computational power because there could be a large number of individual components within the system being modeled (for example, individual cells in an organ).^4^

“ProbRules” is a newer method introduced in 2019 to model a dynamic system that has multiple different scales.^33^ It does this by combining probabilities and logical rules through graph theory: Edge probabilities are initially defined, which are used to derive rules and activities for interactions, and the active rules are then applied. This process is repeated iteratively, with the edge probabilities adjusted each time. The active rules in this model describe the link between levels within the model effectively, as they are independent of the scales involved. It has been demonstrated that ProbRules is effective in simulating the Wnt signaling pathway.^33^

A major area of research in systems biology is tumor development in cancer. If tumor development is considered as a biological system, multiscale modeling can be used to predict outcomes of microscale or macroscale processes and ultimately lead to improved treatment. A more extensive knowledge of the behavior of tumor growth would make precision medicine possible, as specific treatments could be tailored to patients for varying behaviors in tumor growth and development.

### Multiscale cancer modeling, a case study

In their 2009 study, Macklin et al. demonstrated how a multiscale model can represent solid tumor development, revealing how the vascular network is remodeled and how a tumor progresses within each stage of development.^34^ The model considered the varied spatial and temporal scales. For example, the tumor invasion model addresses nutrient transport, tumor cell velocity, and interaction with the microenvironment. Levels within the multiscale model were coupled through their shared variables. As an example, angiogenesis and tumor invasion models were linked through the nutrients released from the vascular network and via the tumor angiogenic factors released from tumor cells. Their results were in accordance with clinical observations, validating the accuracy of the model. This multiscale model also found that when the extracellular matrix is broken down by tumor cells, it dramatically affects the vascular network development and the tumor’s growth response. Additionally, after significant degradation of the extracellular matrix, vessels in the vascular network do not penetrate the tumor, but surround it, meaning nutrient delivery is less effective. These results show that multiscale modeling provides descriptions of the process of tumor development, especially during angiogenesis and vascular growth. Moreover, a potential vulnerability of the tumor is outlined in its lower efficiency of nutrient delivery. This could potentially be further investigated as a target for cancer treatment.

In the same year, a multiscale model for malignant brain tumors was developed by Zhang et al.^35^ In this case, the agent-based modeling (ABM) method was used with a top-down approach: from macroscopic (tumor cells) to molecular (gene expression). The authors discovered information about genes that had elevated expression levels in different tumor cell types. During the analysis, the model was limited because the behavior at the molecular level did not affect the phenotype of the tumor cell at the macroscopic level. To combat this, the authors developed a novel computational model that determined the cancer cell’s phenotype. This was based on experimental data suggesting that a cell either migrates or multiplies but cannot do both at the same time.^35^ The development of a computational model that decides the macroscopic phenotype indicates that multiscale models can still be used when there are gaps in the information of the system. It is possible to predict information at one level given information available from another. This approach has the potential to be very effective in models for systems that are lacking experimental data and were previously unable to be fully modeled. With further development, this type of modeling could contribute to predictive abilities in clinical settings and medicine in general.

Given that the aforementioned studies were published over a decade ago, it should be noted that the field of multiscale modeling has since progressed to focus on optimizing the computation of these models. To this end, a multiscale modeling language was proposed in 2012,^36^ with the aim of making it easier for scientists from various backgrounds to understand the process of developing a multiscale model. There have been numerous software packages developed for computing multiscale models. A systems-biology-specific example is the Enteric Immunity Simulator Multiscale Modeling (ENISIMSM),^37^ which is Java based and was developed for modeling immunological processes. This software can integrate models that use different techniques (such as agent-based, ordinary differential equation [ODE]-based, or PDE-based).^38^ Using this software greatly reduces the time to create a model and would reduce the likelihood of errors during the integration of several models. The wider availability of this software will also make multiscale modeling more accessible and appealing to researchers.

### Human space exploration

**Figure 2:**
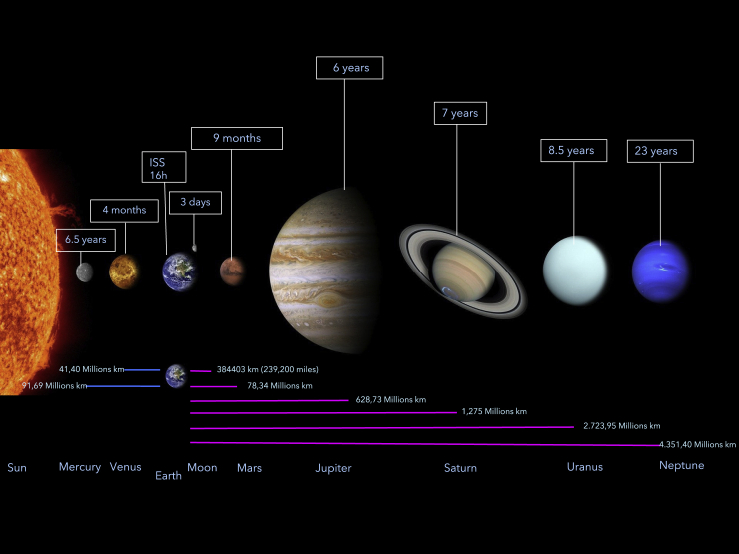
Spatial distance and temporal travel scales from earth Travel time to the International Space Station is approximately 16 h. Travel time to Mars is approximately 9 months. The distance to Mars from Earth varies considerably depending on their orbits around the sun. The closest recorded distance to Mars from Earth was in August 2003 when both were 34.8 million miles (56 million km) apart.

The 20^th^ century saw the very beginning of space missions and the pioneering of human space travel. Originally manifesting because of political competition and lacking true purpose,^39^ it soon evolved into an international cooperation for scientific research.^40^ By 2011, there were 60 active space missions, fueled by both economic and scientific purposes.^41^ The value of human contribution to space travel is paramount due to our skills in rapid sample acquisition, data assimilation and analysis, and pattern recognition.^42^ The planned missions to the Moon and Mars will enable research advances in astronomy, astrobiology, fundamental physics, life sciences, and human physiology and medicine.^43^ Orbital solar power stations and the development of fusion power in space could provide huge amounts of energy to Earth.^44^ Private equity investments in space travel will undoubtedly promote vast economic growth,^45^ and astrotourism is already an emerging industry.^46^ Pragmatically, there are many difficulties with space exploration, given the spatial and temporal travel scales from Earth (Figure 2) and many barriers to human space travel which must be overcome for continued advancements in space travel.

Developing novel strategies to maximize the efficient use of resources such as food, nutrition, and water will be pivotal in long-duration space travel,^47^^,^^48^ along with the development of technologies to combat other detriments to human health such as aging, exposure to microgravity and radiation, as well as psychological stress.

### Cardiovascular system

Commercial spaceflight has evolved rapidly with older persons destined to be exposed to space conditions in the near future. Understanding cardiovascular risk factors and preventive cardiology will be a key component of safe long-term space travel.^49^^,^^50^ Targeted preventive measures, or countermeasures in aerospace medicine, and identifying vascular risks early will be essential in maintaining cardiovascular performance and health during future space missions. The cardiovascular system undergoes major changes in cardiac parameters, from orthostatic intolerance, arrhythmias, and aerobic capacity to cardiac atrophy.^51^ Humans experience a loss in total body fluid mass, and without gravity assisting downward blood flow, there is an anterior shift in body fluid.^52^ Venous blood pooling occurs in the splanchnic, cephalic, and pelvic regions of the body, but it has not yet been determined if this can cause harmful effects to the brain, eye, and splanchnic and pelvic organ morphology or physiology.^53^ Cardiovascular unloading leads to cardiopulmonary deconditioning and, potentially, cardiac atrophy. In addition to diminishing physical activity, this can lead to poorer orthostatic tolerance after return to Earth.^50^ The space environment may directly impact vascular health; however, the clinical relevance of these findings in terms of morbidity and mortality is unclear at this point in time. Van loon and colleagues developed an open-source lumped mathematical model of the cardiopulmonary system that can simulate the short-term adaptations of crucial hemodynamic parameters to an active stand test after being exposed to microgravity.^54^ The model simulates key cardiovascular hemodynamic changes—over a short time frame—during a stand test after prolonged spaceflight under different gravitational and fluid-loading conditions.

### Musculoskeletal system

The musculoskeletal system is highly intricate and shows an acute sensitivity to changes in the biomechanical environment. Microgravity removes gravitational compressive loads in the spine, resulting in swelling of the lumbar intervertebral discs which causes chronic back pain.^55^ It increases bone resorption and reduces bone formation, resulting in the loss of calcium and other bone minerals^56^ causing osteoporosis.^57^ Microgravity also causes rapid skeletal muscle wastage in humans, which is hypothesized to be caused through direct impact on the muscle fibers due to disuse^58^ or indirectly through changes in the levels of factors such as anabolic steroids and growth hormone.^59^ Additional factors such as inflammation, heightened oxidative stress, and calcium dysregulation can also contribute to microgravity-induced muscle loss and weakness.^60^^,^^61^^,^^62^ Microgravity can induce changes in muscle fiber type and composition, which can also alter its function. A study of the effects of prolonged space flight (∼180 days) on the structure and function of slow and fast fibers in human skeletal muscle revealed that prolonged weightlessness caused substantial loss of fiber mass, force, and power with the gradation of the effects being soleus type I > soleus type II > gastrocnemius type I > gastrocnemius type II.^63^ A comparative analysis of the transcriptomes of soleus and extensor digitorum longus (EDL) in mice that underwent 37 days of spaceflight revealed shared stress responses and an altered circadian rhythm. EDL showed more robust growth signals and *Pde2a* downregulation, possibly underlying its resistance to atrophy versus soleus.^13^

### Sodium-retaining endocrine systems

Sodium-retaining endocrine systems are more active in microgravity, resulting in a huge extravascular storage of sodium^64^ and causing degeneration of renal tubular cells.^65^^,^^66^ Changes in the levels of minerals in saliva and urine of astronauts suggest downregulation of parathyroid gland activity.^67^ Research has suggested that long-term exposure to microgravity results in the inhibition of secondary metabolism, but the mechanism needs to be researched further to be fully understood.^68^ At the very least, this would require supplementation of secondary metabolites such as antibiotics. Microgravity also reduces stem and cancer cell proliferation and differentiation, impacting both cancer metastasis and musculoskeletal tissue repair.^69^^,^^70^^,^^71^^,^^72^^,^^73^

### Space radiation

Space radiation is one of the major hazards in human space exploration and poses a serious threat to the integrity of DNA in astronauts.^74^ Galactic cosmic rays contain high (H) atomic number (Z) and energy (E) (HZE) particles that induce extensive DNA damage,^75^^,^^76^ leading to apoptosis, and microgravity has the combined effect of inhibiting DNA repair,^77^^,^^78^ compounding the effects of radiation-induced genomic instability. HZE particle exposure results in altered cognitive function, impaired motor function, and chronic neurological diseases. Ionizing radiation causes oxidizing events within cells, which alter the atomic structure. This can result in defects in mitochondrial physiology, leading to numerous pathological conditions including accelerated aging.^11^^,^^12^^,^^79^ The primary concern of exposure to ionizing radiation is the risk of developing tumors and/or cancer, and the linear no-threshold hypothesis model is implemented to calculate radiation protection.^75^

As noted previously, microgravity impacts the musculoskeletal system. However, ionizing radiation also impacts musculoskeletal responses to microgravity. Deep space travel exposes astronauts to prolonged cycles of ionizing radiation and mechanical unloading, both of which can induce muscle and bone loss. To explore the interactions between skeletal muscle and bone under these conditions, Krause et al. evaluated alterations in the bone and muscle of rodents exposed to hindlimb suspension (HLS) unloading alone or in combination with exposure to proton radiation and radiation of high atomic number (Z) and energy (E) (16O). Their results indicate that combined simulated space radiation and HLS leads to additional bone loss that may not be experienced by muscles.^80^ A limitation of many HLS ground-based models of spaceflight is that the concurrent effects of radiation exposure are not considered, or if they are, the radiation exposure is at a high-dose rate over a very short period. Yu et al. investigated skeletal effects of low-dose-rate gamma irradiation when administered simultaneously to tease out effects solely from disuse caused by HLS.^81^ This revealed that mechanical unloading exhibits greater effects on the bone loss that occurs during spaceflight than low-dose-rate radiation.^81^

Solar particle events, such as coronal mass ejections and solar flares, are characterized by the release of large amounts of high-energy protons by stars in a relatively short period of time. They are temporally unpredictable and can deposit massive doses of radiation in astronauts over a period of as little as a few hours.^82^^,^^83^^,^^84^^,^^85^ Most solar particle events carry enough energy to induce nuclear reactions in the materials they penetrate.^86^ Furthermore, irradiated cells can also transmit radiation-induced effects to neighboring cells through the bystander effect.^87^ These high radiation doses have made solar particle events the predicted primary source of radiation sickness in astronauts on a longer mission outside of Earth’s magnetosphere,^84^ emphasizing their role as a key source of damaging radiation in space.

### Spaceflight injury can be explored using integrated multiomics datasets

To aid the characterization of spaceflight impacts, NASA’s GeneLab database was established. The goal of this repository is to store, curate, and facilitate access to genomic, transcriptomic, proteomic, and metabolomic data from biospecimens flown in space or exposed to simulated space stressors. With multiomics datasets profiling space-flown organisms, this provides researchers with, often preanalyzed, integrable multiomic datasets from humans and model organisms such as *Drosophila melanogaster* and *Caenorhabditis elegans*.^88^ This allows the development of new hypotheses surrounding the impacts of space on human physiology and facilitates new discoveries.^89^

Numerous studies have proven the effectiveness of using integrated multiomics datasets to investigate complex health conditions such as cancer on Earth. Kong et al. analyzed a combined dataset covering genomics, epigenomics, and transcriptomics to conclude that methylation status may have an important role in pancreatic cancer, and they found several potential biomarkers for treatment of pancreatic cancer^90^ Elsewhere, Cao et al. investigated genomic instability through multiomics analysis to find distinct characteristics of risk subgroups for lower-grade glioma, for potential use in prognostic prediction.^91^ Therefore, multiomics modeling provides new insights into cancer biology, which has historically been limited by simpler modeling techniques that can lose accuracy as complexity decreases. In 2016, Liu et al. utilized postanalysis integration of genomic, transcriptomic, and methylomics data to explore mechanisms of DNA repair dysregulation in breast cancer.^92^ This technique could be applied to explore the mechanisms behind DNA repair dysregulation seen under microgravity conditions.^93^ Furthermore, multiomics cancer models integrating genomics, genome conformation, transcriptomics, and epigenomics have been used to predict the phenotypic effects of mutation in cancer,^94^ which could be applied to predict how mutation from spaceflight radiation and cytoskeletal remodeling/DNA modification aberrations due to microgravity affect astronauts.^95^

These techniques can be applied to spaceflight, for example, spaceflight impacts immunity, causing reduced lymphocyte, macrophage, and granulocyte populations,^96^ leading to lowered adaptive immunity, increased aspects of innate immunity, and viral reactivation.^97^ In 2020, Gertz et al. integrated transcriptomic and proteomic data to study how spaceflight affects innate immunity.^97^ They independently analyzed proteomic and transcriptomic data from the NASA Twin’s study, carrying out a pathway interaction analysis on the proteomic data. These findings were validated and further explored using single-cell transcriptomics to enumerate immune cell subtypes and explore the development of monocyte precursors upon return to Earth. They found that the immune impacts of spaceflight may be caused by muscle remodeling which occurs in microgravity and upon return to Earth.^98^^,^^99^ An advantage of this approach is that both RNA and protein data were analyzed, representing 2 heavily interacting omics levels, core to the central dogma of biology.^98^^,^^100^ Furthermore, a separate dataset analysis meant specialist/custom multiomics tools were not needed. Instead, widely available, open-source single-omics tools and R packages, such as Seurat, were used, increasing reproducibility.^29^^,^^98^^,^^101^^,^^102^^,^^103^ Similarly, the proteomic analysis simply repeated an earlier NASA study, allowing the results to be easily double-checked and validated.^98^^,^^104^ A disadvantage of this approach was that the dataset integration relied on human inferences, and thus was subject to observer bias.^29^ Furthermore, this technique does not computationally predict the effects of spaceflight. To predict these impacts, a systems modeling strategy, such as the one described by Shi et al. in 2015, is preferable.^106^

### Mathematical models of terrestrial innate immunity have been developed

Shi et al. developed a system dynamics mathematical model including 18 equations to explain both innate and adaptive immunity.^106^ It encompassed pathogen load, phagocyte activation, and cytokine levels. The advantage of this model is that it considered phagocyte populations and cytokine proteins,^105^^,^^106^ both of which are impacted during spaceflight.^98^ The model is also flexible, allowing for various initial patient conditions^106^ and environmental features of spaceflight to be factored in. This model, however, does not consider important stochastic processes or local interactions such as macrophage movement.^106^ Therefore, it could benefit from being extended to incorporate a deterministic-stochastic cellular automata model that can simulate stochasticity and local cell movements.^107^ Furthermore, Shi et al. only utilized one omics type, proteomics, thus limiting the model’s utility in a systems biology setting.^106^ This model should be expanded to include, at least, transcriptomics because proteomic-transcriptomic studies have provided useful insight into the etiology of spaceflight-associated innate immunity damage.^12^

### Chronic inflammation

**Figure 3:**
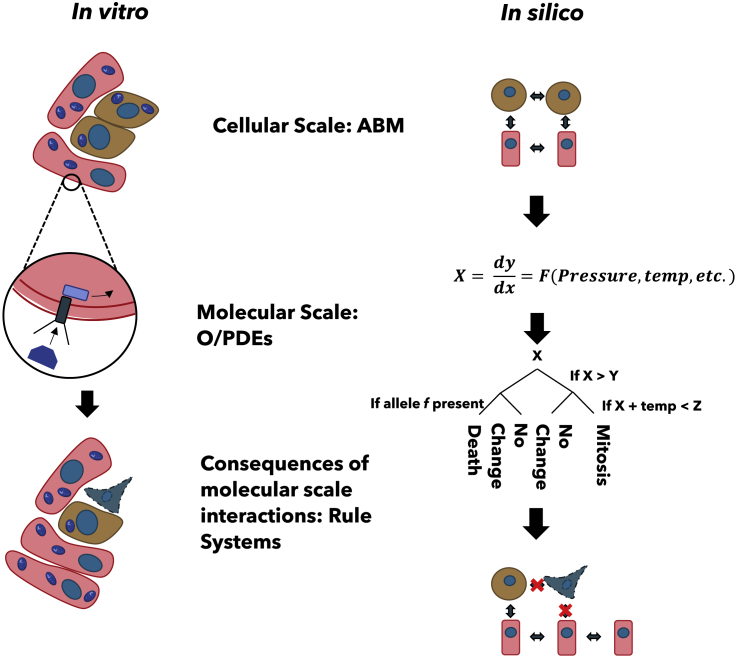
Side-by-side view of an *in vitro* cell environment and a simulation of the same using hybrid multiscale agent-based modeling systems models encompassing ordinary differential equation or partial differential equations (O/PDEs) and agent-based modeling (ABM) Differential equations are used to model chemical interactions at the smallest scale, the results of which are then used in a rule-based agent system to decide the behavior on a cellular scale.

Inflammation is a complex process involving many chemical-receptor interactions and cell states that influence and are influenced by their environment.^108^ Damage from chronic inflammation (CI) occurs at numerous scales from DNA to tissue, causing disease progression.^109^^,^^110^ Therefore, predicting CI impacts requires simultaneous modeling across multiple scales. ABM has been used to study CI, inflammatory processes during sepsis, individual organ infections, and CI diseases.^111^ Cockrell et al. modeled colon inflammation by simulating cell-cell interactions, leveraging signal transduction networks to calculate intercell communication.^112^ Furthermore, transcriptomic data have been utilized in agent-based inflammation modeling, exploring asynchronous granuloma development and the spread of chemokines from infected to uninfected cells.^113^ The ability of MS-ABM models, for example, PhysiCell and PhysiBoSS, to simulate diverse inflammation types and model environmental stress^111^^,^^114^^,^^115^ makes them suitable for predicting the effect of space-associated inflammation as the space environment is an essential component of this inflammation.^116^ High-resolution ABMs are computationally intensive; however, ABMs' performance can be enhanced. To improve performance at smaller scales, a hybrid MS-ABM simulates smaller spatiotemporal events, such as ligand-receptor interactions, using ODEs and PDEs. The results of these ODEs/PDEs are then passed to the agents (cells), which use the results to decide cell behavior^117^ (Figure 3). Furthermore, coarse graining procedures can reduce computational load.^118^

### Cell cycle and circadian rhythm perturbations

Circadian rhythm disturbances during spaceflight are an important factor in sleep disorders commonly seen in astronauts. These sleep disorders can lead to a plethora of issues including social friction between astronauts, reduced energy levels and motivation, and impaired cognition, affecting memory, alertness, and cognitive performance. It is easy to see the sizable impact that such cognitive and social impairment would have in the complex, confined environments on spacecrafts.^12^^,^^119^ Two multiomics studies have uncovered circadian rhythm disturbances in spaceflight. Both articles investigated the transcriptome and proteome, using postanalysis integration via gene ontology terms and gene set enrichment analysis (GSEA) to produce interaction networks.^12^^,^^120^ Furthermore, multiple studies spanning metabolomics, transcriptomics, and proteomics have uncovered cell cycle perturbations during spaceflight.^12^^,^^120^^,^^121^ One study carried out preanalysis integration using MetaboAnalyst, integrating transcriptomic and metabolomic data from NASA GeneLab. The advantage of such preanalysis integration is its avoidance of observer bias as connections between omics datasets are statistically derived instead of being manually inferred.^12^^,^^29^^,^^120^^,^^121^

Although preanalysis and postanalysis integration techniques allow system perturbations to be studied, they do not predict them computationally.^29^ However, protein interaction networks produced during these analyses can facilitate perturbation effect prediction. This process is popular in cancer medicine, for example, patient protein interaction network changes have facilitated prognostic predictions in breast cancer.^122^^,^^123^ Such techniques should, in the future, be applied to space medicine especially given the wealth of information available on multiomics network changes during and after spaceflight.^12^^,^^120^^,^^121^

Mathematical and computational circadian rhythm and cell cycle modeling are both very well established, with models of both processes first appearing in the 1990s.^124^ In 2003, Leloup and Goldbeter produced an early mammalian circadian rhythm model using ODEs which included key circadian rhythm genes and proteins with variable levels of phosphorylation.^125^ This model predicted the effects of day-night cycling in disease. However, it was complex, and entraining it to diseased states proved challenging.^125^^,^^126^ Considered as a classic circadian rhythm model, the model of Leloup and Goldbeter has been expanded to include mathematical parameters representing important circadian-rhythm-associated miRNAs, such as miRNA-219. Although this addition improved the model’s biological representativeness, it increased its complexity.^127^

Increasing complexity makes models more computationally demanding^5^^,^^128^; therefore, improvements like those presented by Liu and Wang^127^ should be approached cautiously to prevent the model becoming prohibitively computationally expensive. However, to apply these models to spaceflight data, further parameters should be included, for example, the effect of mitochondrial dysregulation on circadian rhythms.^12^

### Impaired olfactory functions

Spaceflight affects olfactory functions through unknown etiologies. Suggestions include high CO_2_ concentrations, microgravity, and transcriptomic responses to spaceflight.^129^^,^^130^ Single-omics and multiomics systems biology studies have explored the molecular origins of this olfactory effect.^12^^,^^129^ da Silveira et al. studied proteomic and transcriptomic data via postanalysis integration and found upregulation of olfactory pathways, which also affected circadian rhythm pathways.^12^ However, no studies that included systems models of the olfactory impacts of spaceflight could be found at this time.

Predictive terrestrial olfactory system models have thus far focused on neurons and ligand-receptor interactions.^131^^,^^132^^,^^133^ Although these could be useful in predicting spaceflight effects, they do not consider known transcriptomic changes affecting spaceflight olfaction.^12^ A potential solution for this is intermixing a QM/MM model of olfactory receptor-ligand interactions, as described by Sekharan and colleagues,^132^ with a model of neuronal activation from receptors to the olfactory bulb which considers the transcriptomic status.^134^ Furthermore, a connectome model representing olfactory brain regions (the olfactory bulb), similar to models described by Guntupalli and colleagues, should be included as this model can simulate healthy and perturbed multilayered neuronal structures like the olfactory bulb.^135^^,^^136^ This approach would simulate sensation and nervous transmission to the olfactory bulb while factoring in transcriptomic changes, fully equipping the model to simulate olfaction in spaceflight while reducing computational load using QM/MM.^137^ However, combining 3 models representing a range of scales from molecular to organ region may become extremely complex, leading to extreme computational cost.^128^ However, complexity-reduction techniques could be employed so only those processes involved in spaceflight olfactory damage are modeled.^5^

### Multiomics systems biology is useful but has limitations

Multiomics systems biology modeling is a powerful technique for understanding and predicting the behavior of a system.^4^^,^^5^ However, multiomics models often require hybridizing existing models to link organizational scales, a challenging task requiring much planning, consideration, and model re-evaluation irrespective of component model's quality.^5^ The complexity of these models also requires a lot of computational power, and oftentimes high-performance computing (HPC) infrastructure is essential. Moreover, HPC can take some time to produce solutions.^2^ Furthermore, although predictions can sometimes help test hypotheses, the predictive power of any model is not absolute, as George E.P. Box noted, “all models are wrong, but some are useful”.^138^^,^^139^

Beyond modeling, multiomics integration is also challenging and requires planning, for example, how data are analyzed before integration affects future analyses. Directly comparing datasets at gene/protein level can return confusing results if different samples/datasets are handled or analyzed differently. It is often better to look at more abstracted metrics, for example, running GSEA and comparing enriched terms.^12^^,^^140^

Although more common with the advent of NGS, multiomics integration remains challenging. This is because many analytic techniques designed for single-omics analysis are not suitable for other omics, for example, storage methods used in genomics are not suitable for proteomics. Additionally, if multiomics data are integrated, eventually researchers must carefully separate them back into single-omics datasets before submission to public repositories.^29^

### Future perspectives

Metabolomics has been suggested as a method to overcome the difficulties associated with omics integration. Many metabolomics techniques and analytical tools are compatible with transcriptomics, genomics, and proteomics, and metabolomics is closely associated with organismal phenotype. These factors combined make metabolomics a common denominator across the omics universe; therefore, further metabolomic focus could improve omics integration across systems biology.^29^

Coupling microscales and macroscales is a useful technique but challenging.^4^ Although it has been achieved on the cell-tissue^141^ and pathogen/cell-population scales^142^ for terrestrial environments, no models could be found at this time for spaceflight environments. As shown here, there are terrestrial models that could be used to model spaceflight injuries; however, space is a very extreme environment,^11^ so the efficacy of terrestrial models for spaceflight simulation is dubious. Terrestrial models should be tested for spaceflight simulation efficacy as repurposing existing models would be faster and less resource-intensive than creating new space-specific models.^143^ Such model-scale-coupling, and indeed wider systems modeling, could be facilitated by further development of multiscale modeling software programs, like ENISIMSM, tailored to each type of biological process, or perhaps a more generic software capable of working with various types of systems. However, given that there is not yet a generic mathematical model for systems biology, this will take some time to develop and implement.^2^

Recent multiomics studies have found mitochondria to be a hub of many spaceflight impacts. Therefore, modeling of the mitochondria in space, based on pathways and networks uncovered by da Silveira et al.,^12^ could provide a starting point in predicting adverse spaceflight effects. Although this would be a limited model as it only encompasses 1 organelle, it could serve as the basis for future spaceflight-specific systems biology models. Although significant advances have occurred in space biology over the past decade, additional research is needed to facilitate safer human space exploration. Applied research and the development of countermeasures with a real-time robust biomarker and vitals monitoring is essential to ensure the health of astronauts. Multiomics longitudinal approaches provide key insights into the combined effects of exposures to various space hazards and provide valuable data on interactions between multiple biological features and organ systems.^11^^,^^12^^,^^89^^,^^104^^,^^120^ Each individual aspect of spaceflight biology provides limited insights. Systems analyses encompassing multiple models coupled with human studies will enable a more detailed understanding of physiological and human health-related impacts of the space environment.

### Conclusions

Systems biology techniques integrate multiple biological scales to provide a holistic view of biology.^5^ Multiomics integration is key here as it represents multiple levels of organization^2^ and includes vital contextual information. For example, exposome studies of psychosis with no genomic background would struggle to elucidate why the same exposures lead to disease in some but not in others.^144^^,^^145^ Given the inherent complexity of biological systems, the use of multiscale modeling is essential for accurate and robust simulations. However, there are still no generalized models for multiscale systems, but the wide range of available methods allows for personalized models to be created to best suit the system under investigation.

Although challenging, a well-planned multiomics study can yield a holistic view of a system, facilitating prediction of system perturbation responses.^2^^,^^4^^,^^5^^,^^146^^,^^147^ Such studies have already been used to produce predictive models of biological responses to terrestrial environmental stressors, such as pathogen invasion,^148^ and tumor progression in cancer, which could translate to advancements in the clinical setting, such as optimized tumor imaging.^149^ Extension of these predictive efforts to spaceflight damage is increasingly in-demand as longer, more hazardous missions are planned.^11^^,^^150^ The studies producing these systems models could lead to future breakthroughs in our knowledge of how these systems work. Ultimately, using multiscale modeling of systems biology to simulate the progression of a disease more accurately—for example, solid tumor progression—can aid in optimizing treatments and studying their effects, leading to better health care and potentially precision medicine. Studying spaceflight medicine can help reveal targets and strategies for aging on earth as spaceflight can be considered a rapid aging model. In conclusion, multiscale modeling approaches applied to spaceflight studies will provide new insights and allow development of countermeasures to mitigate against the harsh space environment. Finally, they will guide precision medicine approaches to ensure the health of astronauts during long-term space travel.
